# Deregulation of *MYCN*, *LIN28B* and *LET7* in a Molecular Subtype of Aggressive High-Grade Serous Ovarian Cancers

**DOI:** 10.1371/journal.pone.0018064

**Published:** 2011-04-13

**Authors:** Åslaug Helland, Michael S. Anglesio, Joshy George, Prue A. Cowin, Cameron N. Johnstone, Colin M. House, Karen E. Sheppard, Dariush Etemadmoghadam, Nataliya Melnyk, Anil K. Rustgi, Wayne A. Phillips, Hilde Johnsen, Ruth Holm, Gunnar B. Kristensen, Michael J. Birrer, Australian Ovarian Cancer Study Group, Richard B. Pearson, Anne-Lise Børresen-Dale, David G. Huntsman, Anna deFazio, Chad J. Creighton, Gordon K. Smyth, David D. L. Bowtell

**Affiliations:** 1 Division of Surgery and Cancer, Department of Genetics, Institute for Cancer Research, Oslo University Hospital Radiumhospitalet, Oslo, Norway; 2 Peter MacCallum Cancer Centre, Melbourne, Victoria, Australia; 3 Institute of Clinical Medicine, Faculty of Medicine, University of Oslo, Oslo, Norway; 4 Department of Pathology, University of British Columbia, Vancouver, British Columbia, Canada; 5 Department of Biochemistry, University of Melbourne, Parkville, Australia; 6 British Columbia Cancer Agency, Centre for Translational and Applied Genomics, Vancouver, British Columbia, Canada; 7 Department of Medicine and Abramson Cancer Centre, University of Pennsylvania School of Medicine, Philadelphia, Pennsylvania, United States of America; 8 Department of Gynaecological Oncology, Westmead Hospital, Sydney, New South Wales, Australia; 9 Westmead Institute for Cancer Research, University of Sydney at the Westmead Millennium Institute, Westmead Hospital, Sydney, New South Wales, Australia; 10 Division of Biostatistics, Dan L. Duncan Cancer Center, Baylor College of Medicine, Houston, Texas, United States of America; 11 Walter and Eliza Hall Institute of Medical Research, Parkville, Victoria, Australia; 12 Department of Medicine, Harvard Medical School, Boston, Massachusetts, United States of America; Duke-National University of Singapore Graduate Medical School, Singapore

## Abstract

Molecular subtypes of serous ovarian cancer have been recently described. Using data from independent datasets including over 900 primary tumour samples, we show that deregulation of the *Let-7* pathway is specifically associated with the C5 molecular subtype of serous ovarian cancer. DNA copy number and gene expression of *HMGA2*, alleles of *Let-7*, *LIN28*, *LIN28B*, *MYC*, *MYCN*, *DICER1*, and *RNASEN* were measured using microarray and quantitative reverse transcriptase PCR. Immunohistochemistry was performed on 127 samples using tissue microarrays and anti-HMGA2 antibodies. Fluorescence in situ hybridisation of bacterial artificial chromosomes hybridized to 239 ovarian tumours was used to measure translocation at the *LIN28B* locus. Short interfering RNA knockdown in ovarian cell lines was used to test the functionality of associations observed. Four molecular subtypes (C1, C2, C4, C5) of high-grade serous ovarian cancers were robustly represented in each dataset and showed similar pattern of patient survival. We found highly specific activation of a pathway involving *MYCN*, *LIN28B*, *Let-7* and *HMGA2* in the C5 molecular subtype defined by *MYCN* amplification and over-expression, over-expression of *MYCN* targets including the *Let-7* repressor *LIN28B*, loss of *Let-7* expression and *HMGA2* amplification and over-expression. *DICER1*, a known *Let-7* target, and *RNASEN* were over-expressed in C5 tumours. We saw no evidence of translocation at the *LIN28B* locus in C5 tumours. The reported interaction between *LIN28B* and *Let-7* was recapitulated by siRNA knockdown in ovarian cancer cell lines. Our results associate deregulation of *MYCN* and downstream targets, including *Let-7* and oncofetal genes, with serous ovarian cancer. We define for the first time how elements of an oncogenic pathway, involving multiple genes that contribute to stem cell renewal, is specifically altered in a molecular subtype of serous ovarian cancer. By defining the drivers of a molecular subtype of serous ovarian cancers we provide a novel strategy for targeted therapeutic intervention.

## Introduction

The management of ovarian cancer is in transition. It is increasingly apparent that ovarian cancer is a complex series of distinct tumour types [1], requiring therapies that target molecular features common to the subtypes of the disease. High-grade serous ovarian cancers (HG-SOC) account for a majority of disease-related deaths. Whilst response rates are high to platinum-taxane based adjuvant chemotherapy, there has been little improvement in patient survival over the last decade or more, despite extensive clinical investigation [2]. PARP inhibitors that exploit deficiencies in homologous recombination repair [3], [4] have shown considerable promise, particularly in women with germline *BRCA1* or *BRCA2* mutations. HG-SOC is currently treated as a single entity. A deeper understanding of the molecular drivers of this disease is essential if more effective therapies are to be developed [2]. Recently, gene expression profiling revealed unappreciated diversity within HG-SOC by delineating four distinct molecular subtypes. One subgroup (C1) was defined by a reactive stroma signature, correlating with extensive desmoplasia in such samples. Tumours with the C2 signature were characterised by intra-tumoural infiltration of immune cells, while C4 tumours had a relatively low expression of stromal genes and high levels of circulating CA125. The C5 subtype reflected a mesenchymal cell gene expression signature, and these tumours had sparse immune cell infiltration and were associated with low levels of circulating CA125 [5]. The genetic events that give rise to each molecular subtype of HG-SOC and control their clinical behaviour are currently unknown.

*Let-7*s are a family of twelve sequence-related micro (mi) RNAs distributed over eight genomic clusters that are often down-regulated in cancer [6], [7], [8]. *Let-7* has emerged as part of a complex and important regulatory network in cancer, whose reduced expression leads to re-expression of a range of oncofetal proteins [6], [9]. As with other miRNAs, *Let-7* molecules recognise and bind to their target sequences, resulting in both translational repression and mRNA decay [10], [11], [12]. At a cellular level, *Let-7* has widespread effects on differentiation and self-renewal [13]. The *HMGA2* gene is an extensively characterized target of the *Let-7* family of miRNAs [6], [9], [11], [14] and encodes a DNA binding and chromatin modifying protein that regulates both differentiation and stem cell renewal [15]. High level expression of HMGA2 has also been linked to poor outcome in a range of solid cancers, including ovarian cancer [16]. The balance between *HMGA2* and *Let-7* expression has been tied to maintenance of an undifferentiated state in cancer cells [9], [14]. A negative feedback loop involving high-level expression of the *Let-7* repressors, *LIN28* and *LIN28B*, has been associated with multiple malignancies [14], [17], [18]. The oncogenes c-Myc and N-Myc are positive regulators of *LIN28* and *LIN28B*, respectively [19], [20]. Therefore, deregulation of different aspects of a pathway involving MYC proteins, *Let-7* repressors *LIN28* and *LIN28B*, various *Let-7* alleles, and oncofetal targets such as *HMGA2* have been reported in a range of malignancies.

We have utilized genomic datasets from over 900 HG-SOC to decipher the pathways that control this disease. We show that the C5 subtype of HG-SOC is defined by *Let7* and *MYCN* de-regulation, presenting a new opportunity for targeted therapeutic intervention in ovarian cancer.

## Methods

### Ethics statement

This study was approved by the Human Research Ethics Committees at the Peter MacCallum Cancer Centre, Queensland Institute of Medical Research, University of Melbourne and all participating hospitals. Written informed consent was obtained from all participants in this study.

### Genomic datasets

Microarray gene expression data was obtained from four cohorts, referred to as AOCS, TCGA, NCI and Norway. The AOCS dataset (n = 285) was generated using Affymetrix U133 2.0 arrays and is available at Gene Expression Omnibus (GEO). The Cancer Genome Atlas dataset (TCGA, n = 476) was generated using Affymetrix HTHGU133a arrays, and obtained through the TCGA data portal. The NCI dataset (n = 185) was generated on Affymetrix U133a arrays and obtained from Michael Birrer, Massachusetts General Hospital. The Norway dataset [21], [22] (n = 64) was generated using custom cDNA arrays and obtained through the laboratory of one of us (A.H.). Clinical details for each dataset are summarized in Table S1.

### Patients and samples

Samples for immunohistochemistry and RNA validation studies were obtained from the Australian Ovarian Cancer Study (AOCS), a population-based cohort of women with epithelial ovarian cancer recruited between 2002–2006 [5]. All patients signed an institutionally-approved patient information and consent document. Details of processes for patient accrual, collection of clinical follow up information, pathological review, isolation of nucleic acids, and preparation of tissue microarrays are described previously [5].

### Bioinformatic analyses

A detailed description of the multiple bioinformatic analyses used in this report is provided in Supplementary Methods S1.

### Quantitative real-time polymerase chain reaction (Q-RT-PCR)

Measurement of expression of coding genes was performed as described previously [23], using either TaqMan gene expression assays (Applied Biosystems) or SYBR green (Applied Biosystems). PCR amplification was performed in triplicate for each sample. Endogenous controls *HPRT1* and *ACTB* were included for all assays and relative quantification of mRNA expression was calculated by using the 2^−ΔΔCt^ method [24]. The expression of mature miRNAs for *Let-7* alleles was determined using a TaqMan miRNA Assay (Applied Biosystems) following manufacturer's instructions. Additional details, including primers and cycle times, are provided in Supplementary Methods S1.

### Immunohistochemistry and fluorescence in situ hybridisation

HMGA2 protein expression was measured in HG-SOC samples on a tissue microarray (n = 127) as described in Supplementary Methods S1. Scoring was as follows: 0 - no/weak or moderate nuclear expression, 1 - less than 10% tumour cells with strong nuclear staining, 2 - 10–50% tumour cells with strong nuclear staining, 3 - more than 50% tumour cells with strong nuclear staining. Fluorescence in situ hybridization (FISH) of bacterial artificial chromosome (BAC) probes to metaphase nuclei was as previously described [17].

### Functional assays in cell lines

Knockdown (KD) of target mRNAs was achieved using Dharmacon On-Target Plus Smartpools (Dharmacon, Thermo-Scientific) for all genes except *MYCN*, which was targeted using Qiagen siRNA Hs_MYCN_3 (Qiagen). Controls included Dharmafect1 alone, Dharmacon non-silencing control pool, *GAPDH* smartpool, and All-Stars negative control (Qiagen) for *MYCN* assays. Details of the cell culture and KD transfection conditions are provided in Supplementary Methods S1.

## Results

### Molecular subtypes of HG-SOC

**Figure 1:**
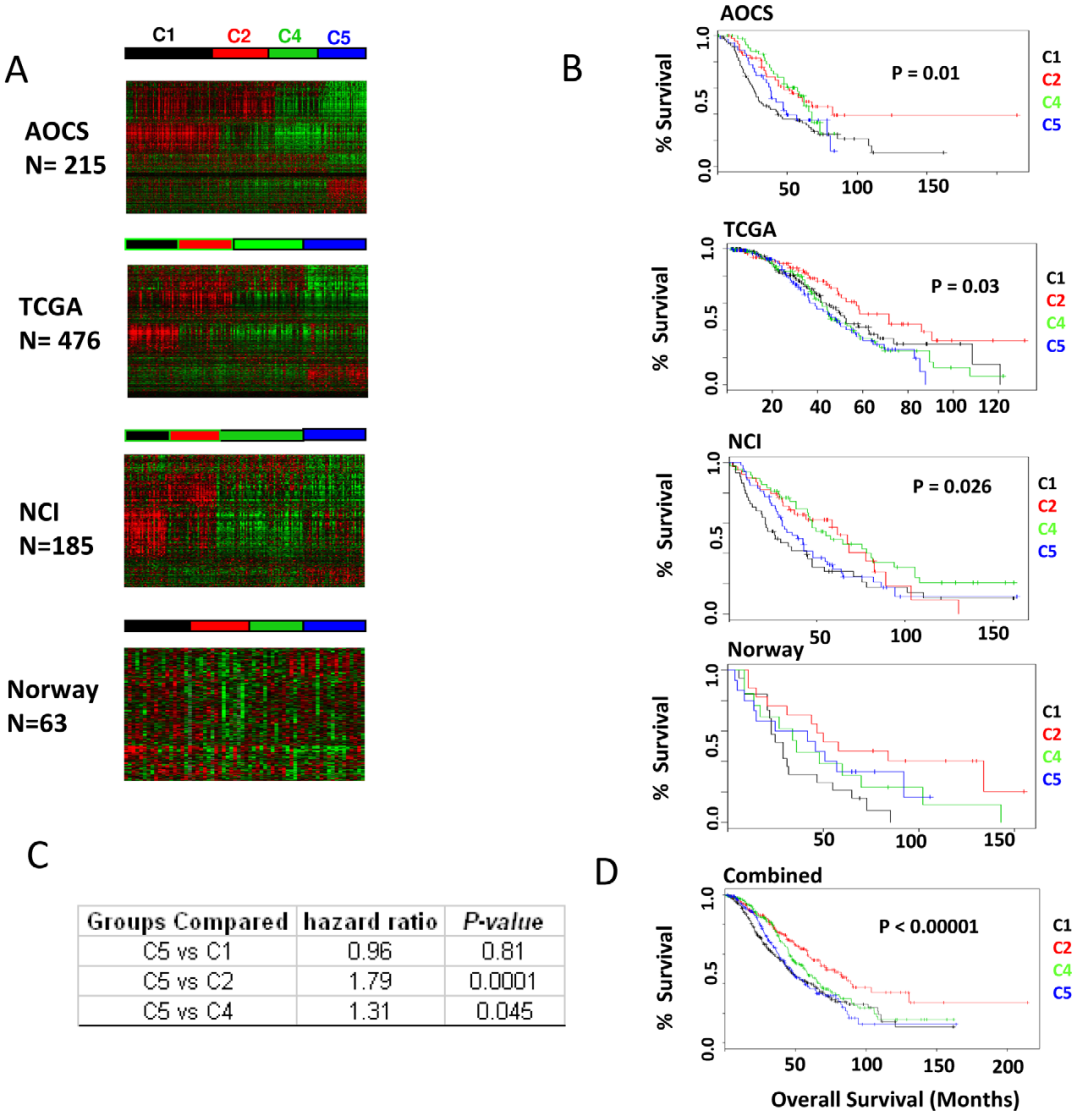
Molecular subtypes of serous ovarian carcinomas with clinical outcome data. (A) Heatmap of gene expression data taken from AOCS, TCGA, NCI and Norway datasets shows that tumours are classified into 4 molecular subtypes. Genes are clustered by Pearson correlation and samples are ordered by molecular subtype. While the original K-means clustering from Tothill et al. is shown for the AOCS cohort, a supervised learning procedure was used for classification of tumours in other datasets (see Supplementary Methods S1). (B) Kaplan-Meier survival curves of samples are plotted. Overall survival is used as the endpoint in all four datasets. Cox proportional hazard model is used to compute statistical significance of the difference in survival between all four groups. Log-rank test p-value is reported. (C) Samples from all datasets were combined to estimate the survival characteristics. Subtypes were compared to C5 and the log rank test p-value given in the table. (D) Kaplan-Meier curves are plotted to depict the survival function of samples in the four different subtypes after combining the samples.

We developed a classifier based on the molecular subtypes (C1, C2, C4, C5) identified in our previous study of 215 tumours from the Australian Ovarian Cancer Study (AOCS) [5]. Using the classifier and a supervised learning procedure, samples were partitioned into one of the four molecular subtypes in datasets from The Cancer Genome Atlas (TCGA) (n = 476), Norway (n = 64) [21], [22] and the National Cancer Institute (n = 185) [25] (Figure 1A, Table S1). Consistent patterns of gene expression and clinical outcome were observed across the datasets. C5 tumours were consistently associated with poor outcome compared with the C2 subtype (Figure 1B–D). We note that there was some variation in the relative frequency of the molecular subtypes in the different datasets and this may be associated with differences in the inclusion criteria used in each study.

### HMGA2 over-expression and Let-7 down regulation

**Figure 2:**
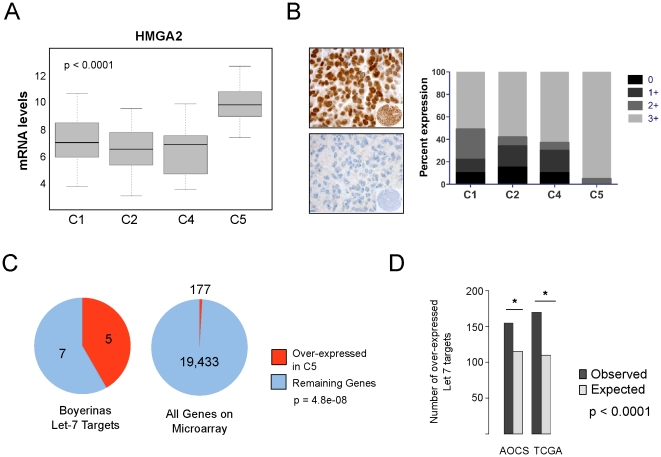
Oncofetal genes deregulated in the C5 subgroup. *Let-7* target genes, including *HMGA2* are specifically deregulated in the C5 molecular subtype of high-grade serous cancers. (A) mRNA expression of *HMGA2* is significantly higher in the C5 molecular subtype. (B) Immunohistochemical analysis of HMGA2 expression in ovarian cancer samples showing consistent over-expression in C5 tumours. Example of strong (3+) staining (top) and no staining (bottom) panel. (C) A core set of *Let-7* regulated target genes (Oncofetal genes) identified by Boyerinas et al [6] are over-represented in C5 gene signature. (D) *Let-7* target genes obtained from TargetScan5.0 are enriched in C5 tumours. The significance of overlap between C5-specific and *Let-7* target gene sets was determined by one-sided Fisher's exact test. Bar plot depicting the association is shown. (* indicates p<0.0001).

To identify subtype-specific pathways we focused on C5 tumours, which are characterised by reduced circulating CA125 immunoreactivity, limited immune cell infiltration, an undifferentiated phenotype [5], and poor overall patient survival (Figure 1B). *HMGA2* is the most strongly over expressed C5-specific gene (AOCS p<0.0001; TCGA p<0.0001; NCI p<0.0001, two sided Mann-Whitney test; Figure 2A, Table S2). Other markers of an undifferentiated phenotype that are highly C5-specific include *DACH1*, *PAX2*, *LAMA1*, *MYCN*, *SOX11*, as well as high-mobility group members *TOX* and *TCF7L1*.

Immunohistochemical analysis also demonstrated that C5 tumours consistently express high levels of HMGA2 protein (∼95% at 3+; p = 0.02, two sided Fisher's exact test; Figure 2B and Table S3). At the protein level, strongly expressing tumours were also present in other subtypes, albeit at a lower frequency. These findings suggest that a specific mechanism of regulating *HMGA2* mRNA expression or stability operates predominately in C5 tumours, and that post-transcriptional mechanisms may influence HMGA2 protein expression in other subtypes. *HMGA2* amplification was more common in C5 tumours (p = 0.005, Mann-Whitney test; Table S4). However, this could not account for the majority of samples showing C5-specific over-expression, as *HMGA2* is significantly over expressed in C5 samples without amplification of *HMGA2* locus (Figure S1). Therefore, while *HMGA2* amplification is more common in C5 tumours, amplification-independent mechanism(s) must account for C5-specific over-expression of mRNA and protein.

*HMGA2* is the most highly predicted target of the *Let-7* family of microRNA (miRNA) in the genome [6], [9], [11], [14], suggesting reduced *Let-7* expression as an alternative explanation for the pattern of *HMGA2* expression in C5 tumours. Consistent with this, we observed C5-specific over-expression of other gene normally repressed by *Let-7*, including a core set of twelve oncofetal genes defined by Boyerinas *et al.* [6] (Figure 2C; p = 4.8×10^−8^, two sided Fisher's exact test). An independently derived gene set of the 100 most highly ranked *Let-7* target genes predicted using a miRNA target prediction model [26] was also significantly enriched in the C5 subtype (p<0.0001, one-sided Fisher's exact test) (Figure 2D).

We then directly measured expression of the *Let-7* family in AOCS samples using a TaqMan assay and compared the findings with miRNA microarray data from the TCGA dataset. We found that *Let-7b*, *-7d*, and *-7i* were significantly under expressed in C5 tumours compared with other subtypes in both the AOCS and TCGA cohorts (Table 1). *Let-7c*, *-7e* and *-7f* were also significantly under expressed in C5 in either TCGA or AOCS datasets. The difference between the two cohorts with these alleles may relate to sensitivity of the assay platform used, miRNA microarrays for TCGA and TaqMan assays for the AOCS samples.

**Table 1:** Differential expression of Let-7 alleles in C5 tumors.

	TCGA data (n = 476)		AOCS data (n = 56)	
	pvalue (fdr)	Log fold change	pvalue (fdr)	Log fold change
hsa-let-7a	0.2	0	0.082	−0.85
hsa-let-7b*	<0.0001	−0.69	0.003	−1.53
hsa-let-7c	0.003	−0.21	0.293	−0.83
hsa-let-7d*	0.008	−0.24	0.009	−1.1
hsa-let-7e	<0.0001	0.49	0.263	−0.46
hsa-let-7f	0.972	−0.04	0.008	−1.17
hsa-let-7g	0.891	0.01	0.087	−0.46
hsa-let-7i*	0.001	−0.25	0.012	−1.28
hsa-miR-98	0.606	−0.14	0.062	−0.81

*Significantly reduced expression.

### Deregulation of Let-7

We explored possible causes of low-level expression of *Let-7* alleles. Genomic deletions, assayed by SNP-based arrays in the TCGA dataset, showed that loss involving the chromosomal region 22q13.31, encompassing *Let-7b* and *Let-7a3*, was more common in C5 tumours (p = 0.018)(Table S5). Although loss of 22q13.31 may account for reduced expression of *Let-7b* in some tumours, it would not account for the effects observed with other *Let-7* alleles, which are distributed over 8 loci and are more likely to be de-regulated in *trans*. Defects in miRNA processing machinery, including reduced levels of Dicer (*DICER1*) and Drosha (*RNASEN*), have been linked to poor outcome in HG-SOC [27], however, higher expression of *DICER1* was observed in the C5 subtype (Figure S2). *DICER1* levels have been shown to be negatively regulated by *Let-7b* [28], [29], potentially explaining the C5-specific over-expression of *DICER1*.

**Figure 3:**
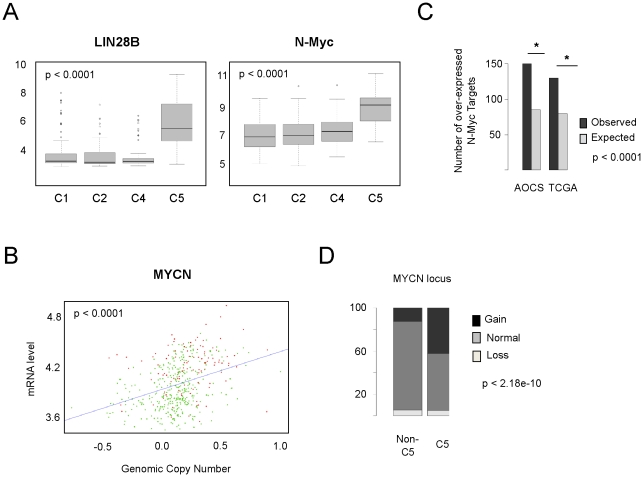
Amplification of *MYCN* and over-expression of *MYCN* and *LIN28B* in C5 tumours. (A) Boxplots depict differential expression of *LIN28B* and *MYCN* in different molecular subtypes of serous ovarian cancers AOCS dataset. (B) DNA copy number levels and expression levels of *MYCN* are highly correlated. C5 samples (coded in red) are predominately distributed in the top right corner of the plot. Samples with segmented copy number log ratio greater than 0.3 were considered to have a gain of *MYCN* and samples with segmented log ratio less than −0.3 were considered to have a loss. Fisher's exact test was used to compute the statistical significance of the association. (C) Association between *MYCN* targets and C5 gene sets. *MYCN* targets were extrapolated from the intersection of two gene lists, (1) genes bound by N-myc in mouse embryonic cell lines and (2) genes with 2-fold increase in expression following transfection of N-myc in mouse embryonic cells (see Supplementary Methods S1). Using data from AOCS or TCGA gene expression sets genes were classified as high or low in C5 tumours versus all other tumours (C1–C4) at p<0.001 by two-sided t-test. The significance of overlap between C5 gene sets and *MYCN* gene sets was determined by a one-sided Fisher's exact test. Bar plot depicting the association is shown.

Lin28 and Lin28B negatively regulate *Let-7* levels by binding to the terminal loops of the precursors of *Let-7* family miRNA, thereby blocking processing into mature miRNAs [17], [30], [31], [32]. We found that the expression of *LIN28B*, but not *LIN28*, was highly enriched in C5 tumours (AOCS p<0.0001, TCGA p<0.0001, two sided Mann Whitney test; Figure 3A and Figure S2). Translocation between *HACE1* and *LIN28B* has been suggested to result in de-regulation of *LIN28B* and result in lowered *Let-7* levels [17]. FISH on a TMA of 239 ovarian tumours, including 73 of known molecular subtype found evidence for rearrangement between *HACE1* and *LIN28B* in only a single core from a HG-SOC tumour of unknown molecular subtype (Table S6), suggesting this mechanism for *LIN28B* up-regulation is rare in ovarian carcinoma.

### Deregulation of *MYCN*

Recently, both c-Myc and N-myc have been shown to positively regulate *LIN28* and *LIN28B* expression [19], [20], however, we found no difference in expression of *MYC* between C5 and non-C5 tumours. Interestingly, gain of *MYC* was more common in non-C5 tumours (p<0.01) (Table S4). By contrast, *MYCN* was significantly over expressed in C5 tumours (AOCS p<0.0001, TCGA p<0.0001, two sided Mann Whitney test; Figure 3A). In the TCGA dataset where both copy number and expression data were available, *MYCN* copy number gain was highly enriched in the C5 subtype (p<0.0001, two sided Mann-Whitney test; Figure 3B). Whilst there was a significant correlation between *MYCN* copy number and gene expression, some C5 samples over expressed *MYCN* without gene amplification, suggesting other mechanisms of subtype-specific regulation. Further evidence of N-Myc activity was obtained by examining expression of known target genes in the AOCS and TCGA cohorts [33]. In both datasets we observed a significant enrichment in expression of N-Myc target genes in C5 tumours (AOCS p<0.0001, TCGA p<0.0001, one-sided Fisher's exact test, Figure 3C), including trans-activation of *LIN28B*. *MYCN* and *HMGA2* expression were also highly significantly correlated (Figure S3). We observed highly specific deregulation of individual members of the *MYCN-Lin28B-Let7* pathway in C5 tumours, as well as a broader set of *MYCN* and *Let7* transcriptional targets. To understand the extent to which the *Let7* pathway defines C5 tumours, we used a recently described signalling pathway impact analysis (SPIA) [34] to probe other signalling pathway dependencies. Amongst 87 signalling pathways tested, *Let7* was the most significantly regulated pathway in C5 tumours (Table S7). Signature genes for all subtypes are presented in Table S8.

### Functional analysis of pathway associations

To explore the functional significance of our findings in primary tumours, we searched genomic data from 40 ovarian cell lines. A2780 and CH1 approximated C5 tumours most closely, as both expressed low levels of *Let-7* alleles, and high levels of *HMGA2* and *LIN28B* (Figure S4A, S4B). However, only CH1 expressed *MYCN*, neither line showed amplification of the *MYCN* locus, and both strongly expressed *LIN28*, the paralogue of *LIN28B* (Figure S4B, S4C). Following systematic knock-down of *LIN28*, *LIN28B* and *MYCN* we monitored expression of the *Let-7* alleles. In both A2780 and CH1 cells, combined siRNA knock-down (KD) of both *LIN28* and *LIN28B* resulted in up-regulation of virtually all Let-7 family members (Figure S5A–B), consistent with previous reports [17], [31]. KD of *LIN28B* was generally not as substantial as *LIN28* (maximum achieved 70% KD, compared to >90% KD) and was associated with less impact on *Let-7* expression. Of particular note, *Let-7* up-regulation following *LIN28/LIN28B* KD was not immediate; despite *LIN28* and *LIN28B* mRNA being suppressed within 48 hours, *Let-7* up-regulation was not detectable until 96 hours. KD of *MYCN* RNA was difficult to obtain (Supplementary Methods S1) in the CH1 cell line. At best a reduction of 60% mRNA expression was achieved (Figure S5B) and little effect on *LIN28B* or *Let-7* expression was observed. Neither KD of *LIN28*, *LIN28B*, nor *MYCN* had a significant effect on HMGA2 protein or mRNA expression (Figure S5C). However, as observed in some C5 tumours, CH1 cells have amplification of *HMGA2* (Figure S4B), suggesting this cell line has an alternative mechanism of HMGA2 over-expression.

## Discussion

**Figure 4:**
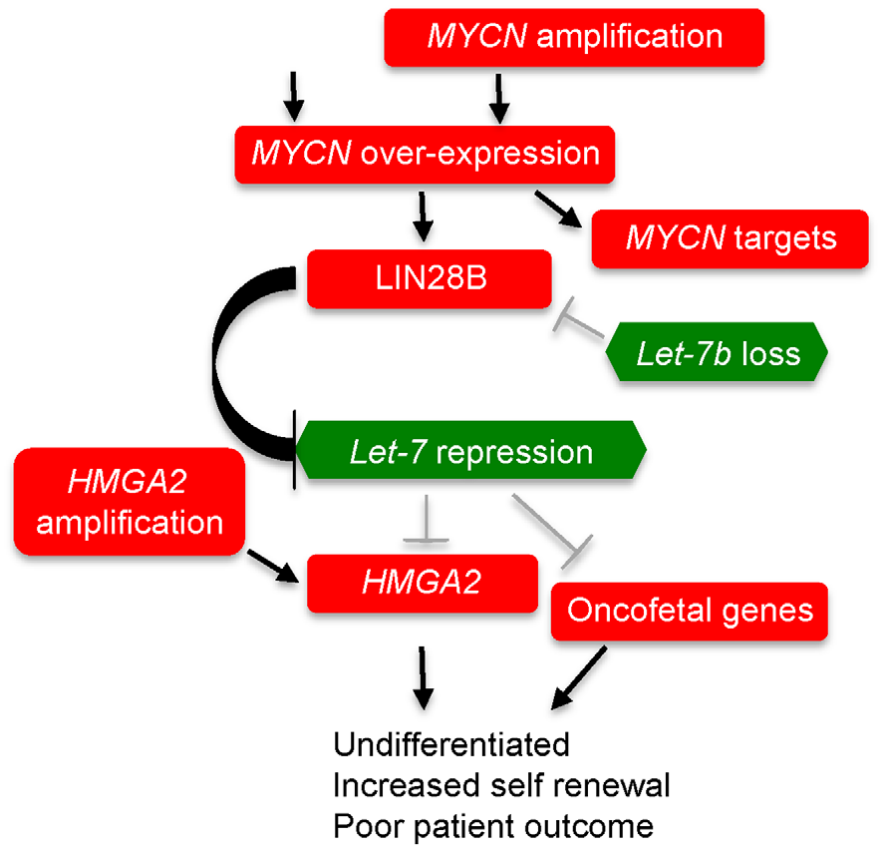
Amplification and over-expression of *MYCN* influences a regulatory loop involving *LIN28B*, *Let-7* and *HMGA2* in C5 high-grade serous tumours. Each event is significantly enriched in C5 versus non-C5 high grade serous tumours: *MYCN* amplification p<0.0001; *MYCN* over-expression p<0.0001; over-expression *MYCN* targets p<0.0001; *LIN28B* over-expression p<0.0001; under-expression *Let-7* alleles p<0.01-0.0001; *HMGA2* amplification p<0.001, *HMGA2* over-expression p<0.0001 (TCGA data). Cumulatively loss of Let-7b, and/or gain of HMGA2, and/or gain of MYCN occur in 77.8% of samples in C5 subtype (p<0.0001, two-sided Fisher's exact test; Table S4).

An extensive series of gain- and loss-of-function experiments in cell lines have demonstrated a functional interaction between N-myc and Lin28B [20]; Lin28B and Let-7 [17], [30], [31], [32]; and Let-7 and HMGA2 and other oncofetal proteins [6]. Here we have shown that each pathway element is specifically expressed in a way that would account for the profound over-expression of HMGA2 and other oncofetal proteins in a large proportion of C5 tumours (Figure 4, Table S9). Gain or over-expression of *MYCN* has not previously been associated with serous ovarian cancer. Whilst a level of amplification is not as high as typically described in neuroblastoma [35], we find significant over-expression of N-myc target genes in C5 tumours supporting the view it is functionally active. Recent data shows that even low-level copy number gain of *MYCN* can significantly influence patient outcome in medulloblastoma [36]. *MYCN* over-expression was highly C5-specific and mechanisms in addition to amplification are likely to contribute to this.

It is likely that several mechanisms lead to *HMGA2* subtype specific expression including *MYCN* amplification, loss of specific *Let-7* alleles including *Let-7b*, and amplification of *HMGA2* itself. For example, in CH1 cells *LIN28* and *LIN28B* are both over expressed and repress *Let-7* expression, however, these cells also show *HMGA2* amplification. We also found a significant association between the C5 molecular phenotype and loss of *Let-7b*. The expression of *Let-7b* was reduced in both AOCS and TCGA datasets, and it is noteworthy that amongst *Let-7* alleles, *Let-7b* has been previously reported as being under expressed in HG-SOC [37]. Although the different alleles of *Let-7* appear to target very similar sequences, the presence of multiple independent genes, their differential expression during development [7] and our data imply they perform selective roles.

The over-expression of *MYCN* and *Let-7* targets in C5 tumours adds weight to the functional significance of the amplification and over-expression of *MYCN* and the significant reduction in expression of *Let-7* alleles. Knockdown of *LIN28* and *LIN28B* expression resulted in re-expression of *Let-7* providing additional evidence of a chain of interactions in ovarian tumours. However, other established interactions were not observed in the ovarian cancer cell lines tested here. For example, although *HMGA2* is a well-defined target of *Let-7* [6], both in gain and loss-of-function experiments, restoration of *Let-7* expression did not noticeably influence *HMGA2* expression. These findings may be explained by limited experimental suppression of *LIN28B* and *MYCN*, the fact that neither CH1 nor A2780 cell lines faithfully phenocopy all of the molecular defects seen C5 tumours, and the amplification of *HMGA2* in CH1 cells. We also note that *Let-7* expression was restored only after an extended period (96 h) of siRNA-mediated knockdown of *LIN28* and *LIN28B*, suggesting that it is difficult to reinitiate the pathway once it has been down-regulated. Further validation of our findings will require cell lines derived from C5 tumours that more closely share the molecular characteristics of their primary counterparts.

Whilst elements of this pathway have been previously demonstrated to be de-regulated in ovarian cancer [37], [38], our report is the first to show complete pathway disruption and its association with a specific subtype of HG-SOC. Our analysis indicates that the *Let-7* pathway is uniquely prominent amongst signalling events disrupted in C5 tumours, and appears to sculpt their transcriptional profile. The identification of molecular subtypes of breast cancer [39], [40] and certain haematological cancers such as diffuse large B-cell lymphoma [41], [42], [43] have provided powerful starting points to discover subtype-specific drivers of disease. Concomitant down regulation of *Let-7* and augmented *HMGA2* expression results in less differentiated tumours with stem cell-like characteristics [6], [9], [13], [14], [15]. These observations are consistent with the low expression of differentiation markers in C5 tumours [5], including *MUC16*, the target of the CA125 antibody used clinically for ovarian cancer diagnosis and prognosis. Our work for the first time defines a pathway in HG-SOC that is associated with and appears to drive the biological and clinical behaviour of a distinct molecular subtype of ovarian cancer, suggesting a targeted therapeutic approach in this group of patients.

## Supporting Information

Figure S1 **HMGA2 gene is significantly up-regulated in C5 tumours from TCGA.** (A) mRNA expression of *HMGA2* based on all samples from TCGA (B) HMGA2 is over-expressed in samples without amplification of HMGA2 locus.
(TIF)

Figure S2 **Expression of a number of C5 specific genes measured using qRT-PCR.** This is done to validate the microarray expression data from the AOCS cohort. Boxplots depicting the relationship between expression levels of these genes and molecular subtype (C5 or Non-C5) are shown, p-values are computed using Wilcox on rank sum test.
(TIF)

Figure S3 **HMGA2 and MYCN expression levels.** (A) HMGA2 and MYCN expression levels are correlated in TCGA samples. (B) HMGA2 and MYCN expression levels are correlated in AOCS samples.
(TIF)

Figure S4 **Cell lines and similarities to the C5 molecular subtype.** A panel of 40 ovarian cancer cell lines was interrogated for similarity to the C5 molecular subtype. (A) Gene expression profiles of *Let-7* alleles in A2780 and CH1 cell lines. (B) Gene expression heatmap of 12 oncofetal genes as well as other defined targets and regulators of the LIN28B-*Let-7* pathway are shown for 40 ovarian cancer cell lines. CH1 and A2780 resemble C5 tumours, with over expression of *HMGA2*, *LIN28B* and *LIN28*. (C) SNP 6.0 Genome-wide copy number profiles of CH1 and A2780. Several key genomic loci are noted: *MYCN*, *MYC*, *HMGA2*, *LIN28* and *LIN28B*. Although neither cell line shows amplification of *MYCN*, CH1 expresses relatively high levels of *MYCN* RNA. CH1 cells also show amplification of *HMGA2*. The relatively limited chromosomal change seen in CH1 and A2780 is atypical of HG-SOC.
(TIF)

Figure S5 **Knock-down results in cell-lines A2780 and CH1.** Heatmaps showing relative knockdown of genes and resulting changes in gene expression in A2780 (A) and CH1 (B). Altered expression of *Let-7* family members was assayed by TaqMan microRNA assays and is displayed over as log2 fold change as per color scale bar. (C) Western blot illustrating change in protein expression following mRNA knockdown of target gene *MYCN* in CH1 cells. Typical experiments are shown. NS, non-silencing control siRNA.
(TIF)

Supplementary Methods S1 **Description of microarray datasets.**
(DOCX)

Table S1 **Summarized clinical annotations for samples from NCI.**
(XLS)

Table S2 **Genes significantly upregulated in C5 subtype.**
(XLS)

Table S3 **Contingency table HMGA2 protein intensity and molecular subtypes.**
(XLS)

Table S4 **Genes significantly gained in C5 subtype.**
(XLS)

Table S5 **miRNA locus significantly lost in C5 subtypes.**
(XLS)

Table S6 **Summary of FISH on TMA for AOCS samples of known molecular subtype.**
(PDF)

Table S7 (XLS)

Table S8 **Genomic aberrations and their association with subtypes.**
(XLS)

Table S9 (XLS)

Table S10 (XLS)
