# Supplementary figures and images for: Deregulation of MYCN, LIN28B and LET7 in a Molecular Subtype of Aggressive High-Grade Serous Ovarian Cancers
Source: PLoS One. 2011 Apr 13;6(4):e18064. doi: 10.1371/journal.pone.0018064 (PMC3076323; doi:10.1371/journal.pone.0018064)

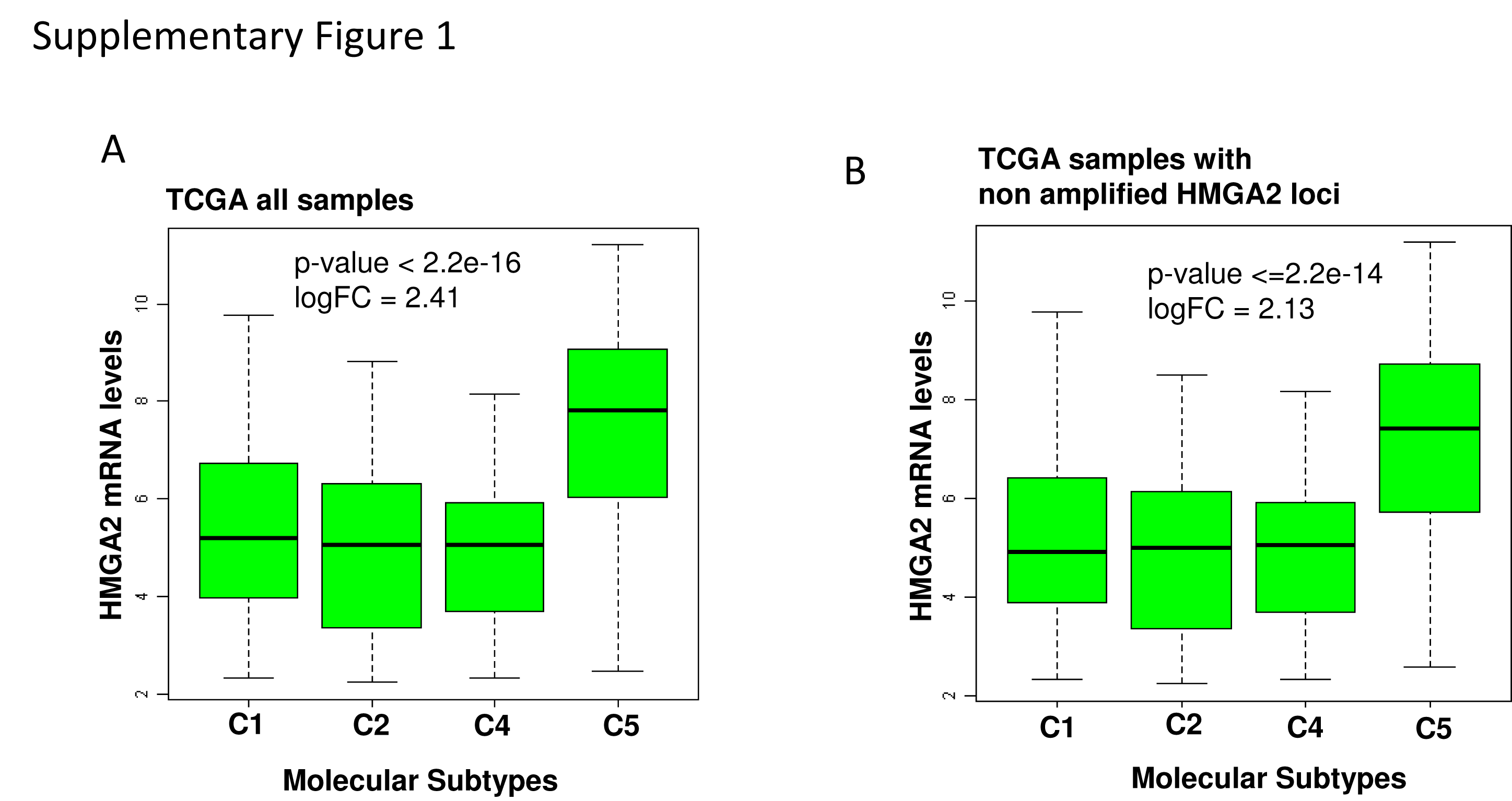

Supplement: Figure S1 — HMGA2 gene is significantly up-regulated in C5 tumours from TCGA. (A) mRNA expression of HMGA2 based on all samples from TCGA (B) HMGA2 is over-expressed in samples without amplification of HMGA2 locus. (TIF) [file pone.0018064.s001.tif]

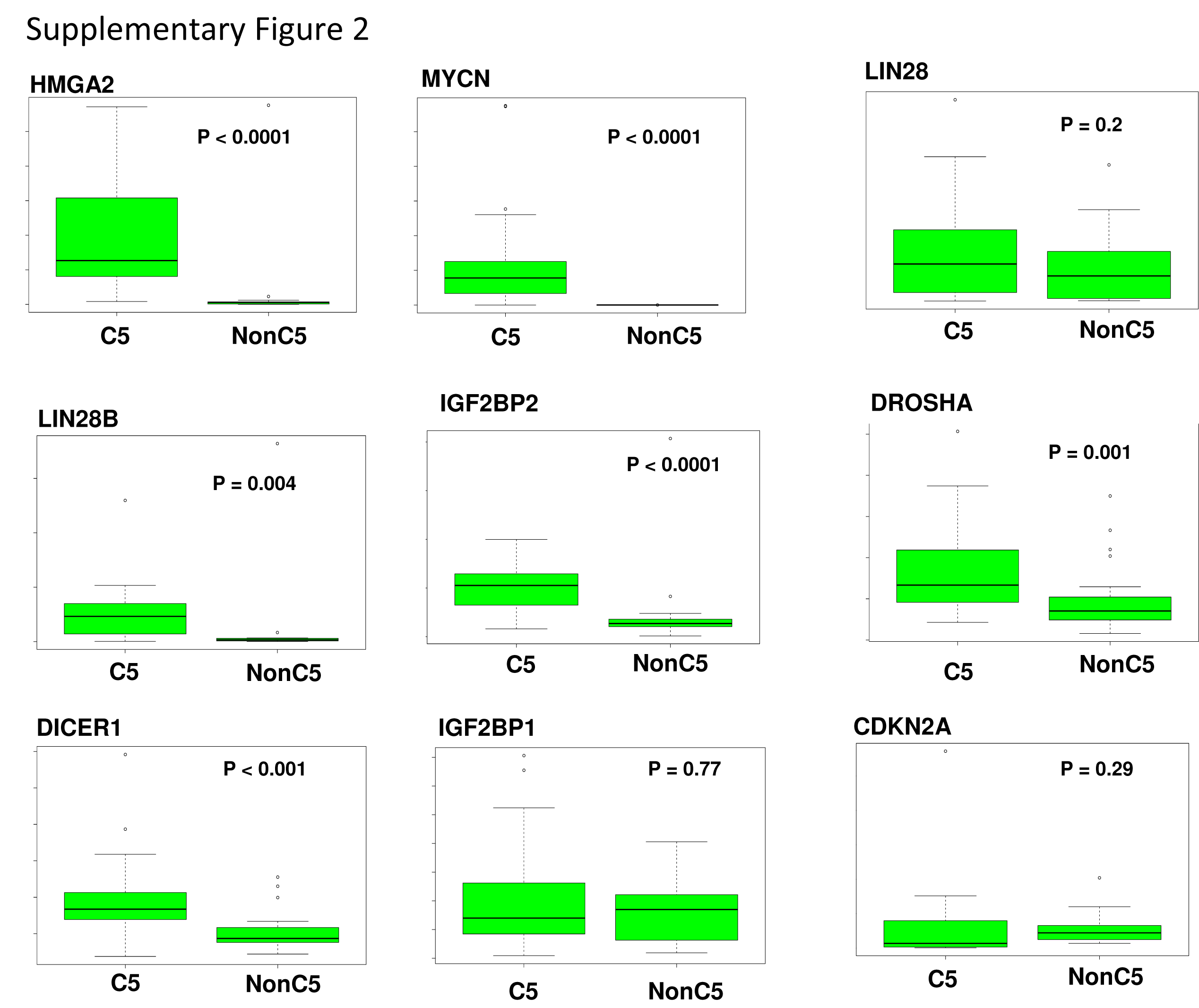

Supplement: Figure S2 — Expression of a number of C5 specific genes measured using qRT-PCR. This is done to validate the microarray expression data from the AOCS cohort. Boxplots depicting the relationship between expression levels of these genes and molecular subtype (C5 or Non-C5) are shown, p-values are computed using Wilcox on rank sum test. (TIF) [file pone.0018064.s002.tif]

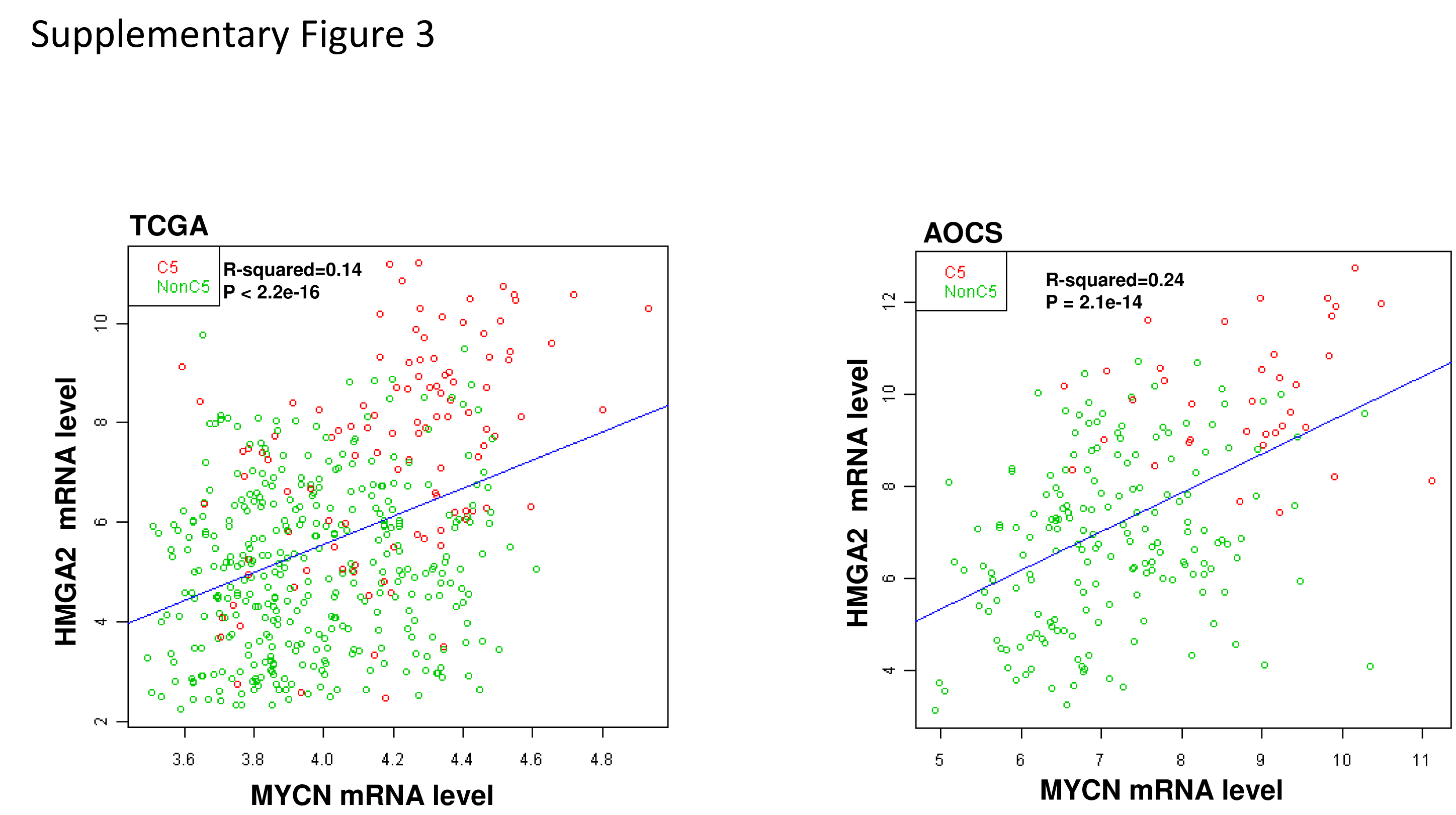

Supplement: Figure S3 — HMGA2 and MYCN expression levels. (A) HMGA2 and MYCN expression levels are correlated in TCGA samples. (B) HMGA2 and MYCN expression levels are correlated in AOCS samples. (TIF) [file pone.0018064.s003.tif]

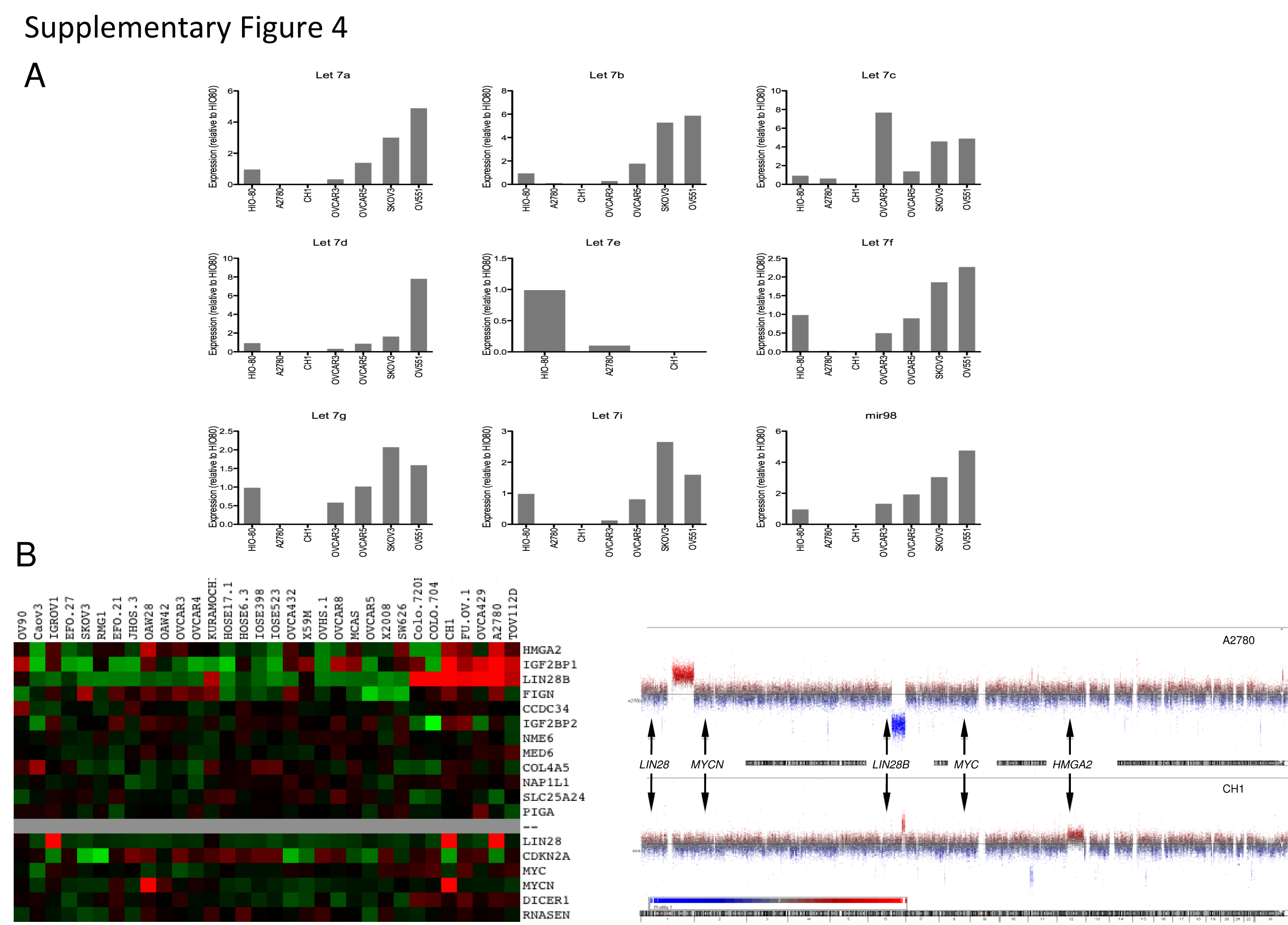

Supplement: Figure S4 — Cell lines and similarities to the C5 molecular subtype. A panel of 40 ovarian cancer cell lines was interrogated for similarity to the C5 molecular subtype. (A) Gene expression profiles of Let-7 alleles in A2780 and CH1 cell lines. (B) Gene expression heatmap of 12 oncofetal genes as well as other defined targets and regulators of the LIN28B-Let-7 pathway are shown for 40 ovarian cancer cell lines. CH1 and A2780 resemble C5 tumours, with over expression of HMGA2, LIN28B and LIN28. (C) SNP 6.0 Genome-wide copy number profiles of CH1 and A2780. Several key genomic loci are noted: MYCN, MYC, HMGA2, LIN28 and LIN28B. Although neither cell line shows amplification of MYCN, CH1 expresses relatively high levels of MYCN RNA. CH1 cells also show amplification of HMGA2. The relatively limited chromosomal change seen in CH1 and A2780 is atypical of HG-SOC. (TIF) [file pone.0018064.s004.tif]

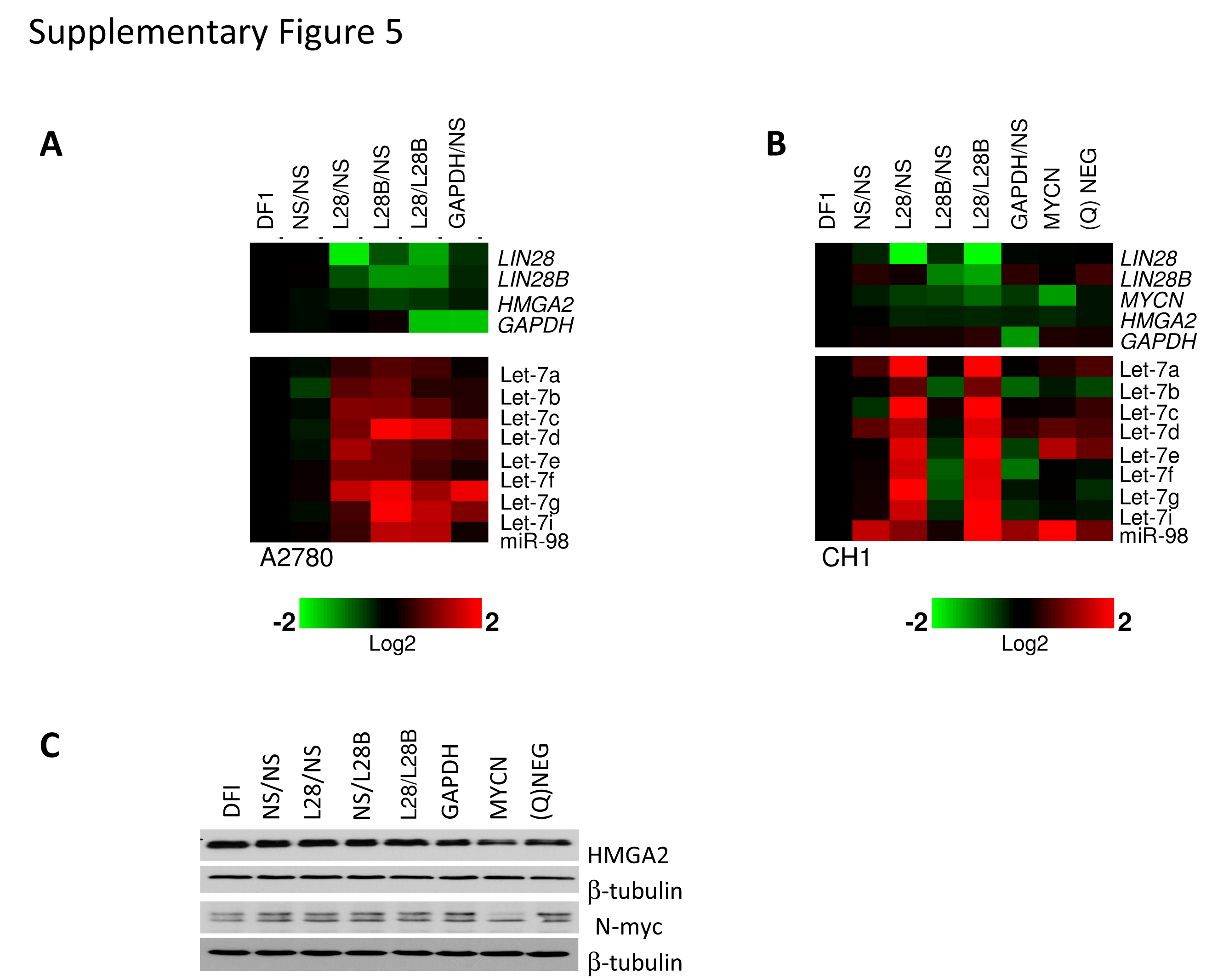

Supplement: Figure S5 — Knock-down results in cell-lines A2780 and CH1. Heatmaps showing relative knockdown of genes and resulting changes in gene expression in A2780 (A) and CH1 (B). Altered expression of Let-7 family members was assayed by TaqMan microRNA assays and is displayed over as log2 fold change as per color scale bar. (C) Western blot illustrating change in protein expression following mRNA knockdown of target gene MYCN in CH1 cells. Typical experiments are shown. NS, non-silencing control siRNA. (TIF) [file pone.0018064.s005.tif]
