# Supplementary material for: Deregulation of MYCN, LIN28B and LET7 in a Molecular Subtype of Aggressive High-Grade Serous Ovarian Cancers
Source: PLoS One. 2011 Apr 13;6(4):e18064. doi: 10.1371/journal.pone.0018064 (PMC3076323; doi:10.1371/journal.pone.0018064)
Supplement: Supplementary Methods S1 — Description of microarray datasets. (DOCX) [file pone.0018064.s006.docx]

# Supplemental Methods:

## Description of microarray datasets

Genomic data of ovarian cancer patients from four different cohorts, referred to as AOCS, TCGA, NCI and Norway are used in this study and are described below. Clinical annotations of all the samples used in the dataset are summarized in Supplementary Table 1.

### AOCS

The ‘AOCS’ data set consists of gene expression data for 285 ovarian cancer patients in the Australian Ovarian Cancer Study project[[1]](#footnote-1). The expression data was previously used to identify molecular subtypes of ovarian cancer ^1^ and is available at Gene Expression Omnibus (GEO)[[2]](#footnote-2).

### TCGA

The Cancer Genome Atlas (TCGA) is a comprehensive and multi-institutional effort to identify the genetics of cancer[[3]](#footnote-3). Molecular data, including miRNA, mRNA, copy number and clinical data for 476 high grade serous samples were obtained through the TCGA data portal (http://cancergenome.nih.gov/dataportal/data/about/).

### NCI

The ‘NCI’ dataset was previously used to identify a gene expression signature predictive of survival in sub optimally debulked ovarian cancer patients ^2^. The dataset consists of 185 high grade serous samples profiled on Affymetrix platform. Data was obtained from Michael Birrer, Massachusetts General Hospital

### Norway

The ‘Norway’ dataset consists of expression data for 64 ovarian cancer samples profiled on cDNA array and described in Chi et al and Murph et al ^3-4^.

## Identification of subtype specific gene signature

The AOCS dataset was used to identify subtype specific gene signature. Sample in the subtype are compared to all samples in other subtypes. Differentially expressed genes are identified using empiric Bayes-moderated *t*-statistics ^5^. The false discovery rate (FDR) was controlled using the Benjamini and Hochberg algorithm. Set of genes with FDR < 0.05 and fold-change > 2 were selected as the signature genes for that subtype.

Set of genes up regulated in C5 and rank ordered based on fold change is shown in Supplementary Table 2.

## Classification of samples in other datasets

Samples in independent datasets are classified into molecular subtypes using the following procedure. For every sample in the dataset, subtype score is computed using a method previously described ^6^. This score is essentially the average log-expression of the signature genes in the sample, weighted by the direction and magnitude of change of those genes in the learning set. Higher score indicate that the transcriptional activity of the sample is similar to that of the subtype. Briefly, subtype score ($sc$) is computed as

$$sc=\sum e(g)*fc(g)$$

Where $e(g)$represents the expression level of the gene in the sample and $fc(g)$ is the log fold change of gene as identified in the gene signature procedure. The sum is taken over all signature genes for that subtype. Subtype scores were evaluated for each sample and each subtype. If multiple probes mapped to the same gene symbol, the probe with the highest average expression was used as the representative probe. Scores are then scaled to have zero mean and unit variance for each subtype within a dataset. As higher value of the score indicates that the transcriptional activity of the sample is similar to the subtype, samples are classified into the subtype with maximal score.

Signature genes for all subtypes are presented in Supplementary Table 9.

## Clinical validation of the classification procedure

The above classification procedure was applied to three different datasets (TCGA, NCI and Norway). Heat maps depicting the expression profile of samples across subtypes are shown in Fig. 1A. Samples are not clustered but genes are clustered using correlation as a similarity measure and average linkage agglomeration method. Kaplan Meier survival curves are used to compute association between molecular subtypes and clinical outcome and are shown Fig. 1B. Statistical significance is computed using Cox proportional hazards model and log rank test p-value is reported. Samples from all the cohorts are combined and the survival characteristics of all groups were compared to C5. Cox proportional hazard model was fitted to estimate the significance of survival difference between C5 and the remaining subtypes and is given in Fig. 1C. Kaplan-Meier curves are shown to depict the survival characteristics of all groups in Fig. 1D.

## Association of Let-7 inactivation status with C5 subtype

The C5 signature included 182 significantly up regulated genes. HMGA2, a direct target of Let-7 miRNA, was the top ranked gene in this set. Boxplots depicting the relationship between HMGA2 expression and C5 subtype are shown in Fig. 2A.

A number of Let-7 regulated oncofetal genes were also present in the C5 signature gene set. Pie chart’s depicting the overrepresentation of oncofetal genes in the C5 signature is depicted in Fig. 2C. Five (HMGA2, LIN28B, IGF3BP2, COL4A5 and FIGN) out of the twelve oncofetal genes were members of the C5 signature set. Fisher’s exact test was used to compute association between oncofetal genes and the C5 subtype.

Conserved Let-7 targets were obtained from TargetScan. A less conservative C5 specific gene set was obtained by comparing the expression profile of C5 tumors versus all other tumors (C1-C4) at p<0.001 by a two-sided t-test. The significance of overlap between C5 gene sets and Let-7 target gene sets was determined by a one-sided Fisher’s exact test. Bar plot depicting the association is shown in Fig. 2D.

C5 signature genes also contain LIN28B, a negative regulator of Let-7b, and MYCN, which is known to regulate LIN28B. Expression levels of these two genes are also specifically associated with C5 subtype and are depicted as boxplots in Fig. 3A. Statistical significance is computed using a two sided Mann-Whitney test.

To verify transcriptional activity of MYCN, we computed the association between MYCN targets and C5 gene sets. MYCN targets were extrapolated from the intersection of two gene lists, (1) genes bound by N-myc in mouse embryonic cell lines^7^ and (2) genes with 2-fold increase in expression following transfection of N-myc in mouse embryonic cells^8^. Using data from AOCS or TCGA gene expression sets genes were classified as high or low in C5 tumors versus all other tumors (C1-C4) at p<0.001 by a two-sided t-test. The significance of overlap between C5 gene sets and MYCN gene sets was determined by a one-sided Fisher’s exact test. Bar plot depicting this association is shown in Fig. 3C.

Summary statistics for all Let-7 and MYCN targets are presented in Supplementary Table 10.

Signaling pathway impact analysis^9^ which measures the actual perturbation of a pathway was used to identify molecular pathways that are inhibited or activated in C5 subtypes. Let7 pathway was constructed to include MYCN and all the remaining 12 oncofetal genes^10^. MYCN was considered upstream of Let7 and the oncofetal genes the downstream targets of Let7. This pathway along with the remaining KEGG pathways was tested for their association with C5 subtype. Results of this analysis are given in Supplementary Table 7.

## Immunohistochemical analysis

HMGA2 protein expression was measured on a subset of samples (n=127).

Formalin-fixed, paraffin-embedded (FFPE) tissue section (4 µm) were stained using the Dako EnVision™ Flex+ System (K8012, Dako Corporation) and Dakoautostainer. Sections were deparaffinized and epitopes unmasked using PT-Link (DAKO) and EnVision™ Flex target retrieval solution, high pH. After treatment with 0.03% hydrogen peroxide (H_2_O_2_) for 5 minutes to block endogenous peroxidase, the sections were incubated with polyclonal rabbit antibody HMGA2 (cat. no.59170AP, 1:500, Biocheck, Inc.) overnight at 4^o^C. The specimens were then given a sequential incubation with EnVision™ Flex+ Rabbit linker (15 min) and EnVision™ Flex/HRP enzyme (30 min). Tissue was stained for 10 minutes with 3`3‑diaminobenzidine tetrahydrochloride (DAB) and then counterstained with haematoxylin, dehydrated and mounted in Diatex. All series included positive controls, which consisted of human ovarian carcinomas that had been shown to express HMGA2. The immunoreactivity was evaluated according to the number of positively stained tumour cells and the intensity of staining. Samples with no/weak or moderate nuclear expression were scored as negative (0), less than 10% tumour cells with strong nuclear staining were scored as low expression (1), 10-50% tumour cells with strong nuclear staining were scored as intermediate expression (2) and more than 50% tumour cells with strong nuclear staining were scored as high expression (3).

Bar plot depicting the relationship between protein expression and molecular subtypes is shown in Fig. 2B. Even though a high level of HMGA2 protein expression is present in a substantial fraction of high grade serous tumours, C5 tumours are enriched for high HMGA2 protein (Supplementary Table 3). Association between molecular subtypes and HMGA2 protein expression was tested using a Fisher’s exact test.

## Validation of Let-7 inactivation in C5

Expression levels of Let-7 family of genes were measured using a Taqman assay on a number of randomly selected samples in the AOCS cohort. Differential expression was tested using two sided Wilcoxon Rank Sum test. The false discovery rate (FDR) was controlled by using the Benjamini and Hochberg algorithm. False discovery rate and median fold change for all the tested miRNA’s are given in Table 1.

Normalized (Level 3) miRNA array data for all TCGA samples were downloaded from the TCGA data portal. TCGA samples were classified into molecular subtypes using a supervised learning method (described below). Expression level of Let-7 family miRNA levels were compared between samples belonging to C5 and the other subtypes using a two sided Wilcoxon-rank sum test. The false discovery rate (FDR) was controlled by using the Benjamini and Hochberg algorithm. False discovery rate and median fold change for all the tested miRNA’s are given in Table 1.

## Copy number changes in C5 subtype

Genome wide copy number changes were compared to identify genes specifically amplified in C5 subtype tumors compared to non-C5 tumors. Statistical significance of association between amplification status and subtype was tested using a two sided Fisher’s exact test. False discovery rate was controlled by using the Benjamini and Hochberg algorithm. The results for all the genes are shown in Supplementary Table 4 and Fig. 3B for the MYCN locus.

A similar procedure was used to assess the association between loss of loci containing Let-7 family genes and molecular subtypes as shown in Supplementary Table 5.

The three molecular events; amplification of MYCN locus, amplification of HMGA2 and loss of Let-7b locus were all significantly associated with C5 subtype. Proportion of samples in C5 and other subtypes with these events are reported in supplementary Table 8. Statistical significance of association was tested using a two sided Fisher’s exact test.

## Copy number change and mRNA levels

To test the possibility of MYCN copy number driving MYCN expression levels we correlated segmented value of MYCN locus to MYCN mRNA levels for all samples in the TCGA cohort. Scatter plot depicting the relationship is shown in Fig 3B. C5 samples (plotted in red) are in the top right corner of the plot suggesting that these samples are enriched for gain at MYCN loci and have high level expression of MYCN.

HMGA2 locus was also amplified in C5 subtype albeit at a lower frequency. HMGA2 expression level is specifically associated with C5 subtype (Supplementary Fig. 1A). Even after removing HMGA2 locus amplified samples the relationship between HMGA2 and C5 subtype was very highly significant (Supplementary Fig. 1B). Significance of association was computed using a two sided Mann-Whitney test.

Transcript levels of HMGA2 and MYCN was also significantly correlated in both AOCS and TCGA dataset as shown in (Supplementary Fig. 3).

## Fluorescence in Situ Hybridization

FISH was conducted using standard protocols on metaphase nuclei as previously described^11^. Source material consisted of 6uM sections from FFPE TMA from the AOCS. The RP-770C15 and RP-809N15 BAC probes were obtained from the RPCI-11 human BAC library and directly labeled with SpectrumGreen and SpectrumRed, respectively, via a nick translation kit (Vysis), hybridized in a humidified chamber overnight at 37 °C, and counterstained with 4,6-diamidino-2-phenylindole. Detection was conducted using a Zeiss Axioplan epifluorescence microscope equipped with a COHU-CCD camera and Quips software.

Summary data for FISH on TMA for AOCS samples are shown in supplementary Table 6. Overall a low proportion of tumours showed molecular changes by FISH, a number of C5 and non-C5 tumours did show gain for the region encompassing *LIN28B* and *HACE1* in at least one examined core. However, heterogeneity between cores from the same samples was common, and for tumours of known molecular subtype, no association of gain or loss at the *HACE1*/*LIN28B* loci was attributable to C5 tumours (Supplementary Table 6).

## Primary Samples for quantitative analysis of Let-7 expression by TaqMan assay

38 primary serous ovarian tumours representing different subtypes were selected from the cohort described in Tothill et al, including 20 samples from the C5 and 18 samples from other subtypes. Expression levels of these genes are summarized as boxplots in Supplementary Fig. 4. Wilcoxon rank sum test was used to compute statistical significance of differential expression.

## RNA isolation

Total RNA (including mRNA and miRNA) for mRNA and miRNA assays was isolated using the mirVana RNA isolation kit (Applied Biosystems). RNA concentrations and quality were determined using NanoDrop spectrophotometer and the Agilent RNA 6000 Bioanalyser respectively.

## Quantitative Real-Time RT-PCR

Total RNA (1μg) was reverse transcribed prior to RT-PCR using M-MLV reverse transcriptase. RNA was denatured with an equal amount of random hexamer primers at 70°C for 5 minutes then cooled on ice to anneal. Synthesis of cDNA was performed using 200U M-MLV reverse transcriptase and 12.5nmol each dNTP in reaction buffer (Promega) at 42°C for 90 minutes followed by a 95°C enzyme deactivation step for 5 minutes. Evaluation of mRNA by qRT-PCR was performed using either TaqMan gene expression assays with TaqMan Master Mix (Applied Biosystems PN# 4367846) or SYBR green using SYBR PCR master mix (Applied Biosystems PN# 4309155). PCR amplification was performed in triplicate for each sample on an ABI 79OOHT following 2-step 40 cycle standard PCR conditions for TaqMan assays. Endogenous control for *HPRT1* and *ACTB* were included for all assays and relative quantification of mRNA expression was calculated by using the 2^-∆∆Ct^ method.

*Primers:*

| **Target** | **Primer Name** | **Sequence (5’ - 3’)** | **[Final] in PCR (pM)*** | **Applied Biosystems Assay ID** |
| --- | --- | --- | --- | --- |
| ***LIN28*** | NM024674_LIN28_F | AGGAAAGAGCATGCAGAAGC | 100 |  |
|  | NM024674_LIN28_R | GCTACCATATGGCTGATGCTC | 100 |  |
| ***LIN28B*** | NM001004317_LIN28B(3p)_F | TCTTCCAAAGGCCTTGAGTC | 100 |  |
|  | NM001004317_LIN28B(3p)_R | ATGATGATCAAGGCCACCAC | 100 |  |
| ***LIN28*** |  |  | 1x | Hs00702808_s1 |
| ***LIN28B*** |  |  | 1x | Hs01013729_m1 |
| ***HMGA2*** | 8091_HMGA2_F | ACTTCAGCCCAGGGACAAC | 100 |  |
|  | 8091_HMGA2_R | CTTGTTTTTGCTGCCTTTGG | 100 |  |
| ***HMGA2*** |  |  | 1x | Hs00171569_m1 |
| ***HPRT1*** | 285_HRPT_L | GTTATGGCGACCCGCAG | 100 |  |
|  | 285_HRPT_R | ACCCTTTCCAAATCCTCAGC | 100 |  |
| ***ACTB*** | 283_ACTB_L | GCACAGAGCCTCGCCTT | 100 |  |
|  | 284_ACTB_R | GTTGTCGACGACGAGCG | 100 |  |
| ***MYCN*** | 4613_MYCN_F | CACAAGGCCCTCAGTACCTC | 100 |  |
|  | 4613_MYCN_R | ACCACGTCGATTTCTTCCTC | 100 |  |
| ***GAPDH*** | 273_GAPDH_L | AAGGTGAAGGTCGGAGTCAA | 100 |  |
|  | 274_GAPDH_R | AATGAAGGGGTCATTGATGG | 100 |  |
| ***ACTB*** |  |  | 1x | Hs99999903_m1 |
| ***CDKN2A*** |  |  | 1x | Hs00923894_m1 |
| ***RNASEN (Drosha)*** |  |  | 1x | Hs00203008_m1 |
| ***DICER1*** |  |  | 1x | Hs00229023_m1 |
| ***IGF2BP1 (IMP1)*** |  |  | 1x | Hs00198023_m1 |
| ***IGF2BP2 (IMP2)*** |  |  | 1x | Hs01118009_m1 |
| **for TaqMan gene expression assays primer probe mix is diluted to 1x from 20x stock* | | | | |

## TaqMan assays for Let-7 family members

Expression of mature miRNAs (Let-7 family) were determined using a TaqMan miRNA Assay (Applied Biosystems) following manufacturer’s instructions. Briefly, single-stranded cDNA was synthesized from 10 ng of total RNA in a 15 ul reaction volume using the TaqMan MicroRNA Reverse Transcription kit (Applied Biosystems). Each Let-7 or control cDNA synthesis reaction was performed separately. Samples were incubated at 16°C for 30 min followed by 42°C for 30 min and inactivated at 85°C for 5 min. Quantitative PCR for each cDNA was done in triplicate on an Applied Biosystems 7900HT sequence detection system (Applied Biosystems). Small RNA RNU6B or U47 were used as endogenous controls and run for each sample/assay. Relative quantification of miRNA expression was calculated using the 2^-∆∆Ct^ method.

 TaqMan microRNA assays:

| ***Assay Target*** | ***TaqMan Assay ID (Applied Biosystems)*** |
| --- | --- |
| *hsa-let-7a* | *2307* |
| *hsa-let-7b* | *2404* |
| *hsa-let-7c* | *2405* |
| *hsa-let-7d* | *2283* |
| *hsa-let-7e* | *2407* |
| *hsa-let-7f* | *382* |
| *hsa-let-7g* | *2118* |
| *hsa-let-7i* | *2172* |
| *hsa-miR98* | *577* |
| *RNU6B (endogenous control)* | *1093* |
| *U47 (endogenous control)* | *1223* |

## Cell Culture

A2780 and CH1 cells were graciously provided by the laboratory of Professor Ian Campbell. Both cell lines were propagated in RPMI1640 supplemented with 10% Fetal Calf Serum in a humidified incubator at 37°C with 5% CO2. Cells were passaged 1:10 approximately every four days.

## siRNA Knockdown experiments.

Knockdown (KD) of target mRNAs was done using Dharmacon On-Target Plus Smartpools (Dharmacon, Thermo-Scientific) for all genes of interest except *MYCN*. Transfections were done with controls including Dharmafect1 (DF1; lipid alone), Dharmacon non-silencing control pool (NS) and smartpool for *GAPDH* in each experiment. For experiments including *MYCN* knockdown Qiagen siRNA Hs_MYCN_3 (Qiagen; SI00076300) and the Qiagen all-stars negative control (Qiagen; 1027280) were used, in addition to the control listed above.

Unless otherwise noted in the text cells were seeded at 5x10^4^ cells/ml (CH1) or 1x10^5^ cells/ml (A2780) on day zero (0). Cells were transfected on day 1 as per manufacturers instruction using DharmaFECT1 (DF1; 0.4% v/v) and 50nM gene specific or control siRNA. For experiments involving simultaneous knockdown of two genes 50nM gene1 (or NS) siRNA and 50nM gene2 (or NS) siRNA were co-transfected. Media was changed on day 2 (24hrs post transfection), and cells were assayed for KD at 96hrs post transfection (day 4). Experiments for which RNA or protein was collected were scaled exactly to a 12-well plate format with 1ml media.

## Western Blot

Whole cell lysates were collected in Laemli buffer by direct lysis of cells on tissue culture plates. Lysates were boiled, resolved by SDS-PAGE using 10% (w/v) acrylamide gels and transferred to PVDF membranes (Millipore). Blots were blocked in 5% Skim-Milk powder diluted in phosphate buffered saline with 0.1% Tween-20 (PBST). Primary antibody was diluted in PBST and incubated overnight with gentle agitation at 4°C. Blots were washed and incubated with HRP-conjugated secondary antibody for 1 hour at room temperature followed by washing in PBST, and developed with chemiluminescent substrate (ECL-plus; Amersham Biotechnology). Detection was done via exposure to radiographic film. Primary anti-bodies used include anti-N-myc (Dilution 1:500; ID# sc-53993; Santa Cruz Biotechnology), anti-HMGA2 (Dilution 1:500; ID# H00008091-M02; Novus Biologicals). Secondary antibody – HRP-conjugated donkey–anti-mouse (1:10,000, ID# 715-035-150 Jackson ImmunoResearch). Blots were re-probed (as above) with an antibody against α-tubulin (Dilution 1:7,000; ID# T-5168; Sigma-Aldrich) to assess protein loading.

## References for Supplemental Material:

1 Tothill, R. W. *et al.* Novel molecular subtypes of serous and endometrioid ovarian cancer linked to clinical outcome. *Clin Cancer Res* **14**, 5198-5208, doi:14/16/5198 [pii]

10.1158/1078-0432.CCR-08-0196 (2008).

2 Bonome, T. *et al.* A gene signature predicting for survival in suboptimally debulked patients with ovarian cancer. *Cancer research* **68**, 5478-5486, doi:68/13/5478 [pii]

10.1158/0008-5472.CAN-07-6595 (2008).

3 Chi, J. T. *et al.* Gene expression programs in response to hypoxia: cell type specificity and prognostic significance in human cancers. *PLoS Med* **3**, e47, doi:05-PLME-RA-0452R2 [pii]

10.1371/journal.pmed.0030047 (2006).

4 Murph, M. M. *et al.* Lysophosphatidic acid-induced transcriptional profile represents serous epithelial ovarian carcinoma and worsened prognosis. *PLoS One* **4**, e5583, doi:10.1371/journal.pone.0005583 (2009).

5 Smyth, G. K. Linear models and empirical bayes methods for assessing differential expression in microarray experiments. *Stat Appl Genet Mol Biol* **3**, Article3, doi:10.2202/1544-6115.1027 (2004).

6 Lim, E. *et al.* Aberrant luminal progenitors as the candidate target population for basal tumor development in BRCA1 mutation carriers. *Nat Med* **15**, 907-913, doi:nm.2000 [pii]

10.1038/nm.2000 (2009).

7 Marson, A. *et al.* Connecting microRNA genes to the core transcriptional regulatory circuitry of embryonic stem cells. *Cell* **134**, 521-533, doi:S0092-8674(08)00938-0 [pii]

10.1016/j.cell.2008.07.020 (2008).

8 Chen, X. *et al.* Integration of external signaling pathways with the core transcriptional network in embryonic stem cells. *Cell* **133**, 1106-1117, doi:S0092-8674(08)00617-X [pii]

10.1016/j.cell.2008.04.043 (2008).

9 Tarca, A. L. *et al.* A novel signaling pathway impact analysis. *Bioinformatics* **25**, 75-82, doi:btn577 [pii]

10.1093/bioinformatics/btn577 (2009).

10 Boyerinas, B. *et al.* Identification of let-7-regulated oncofetal genes. *Cancer Res* **68**, 2587-2591, doi:68/8/2587 [pii]

10.1158/0008-5472.CAN-08-0264 (2008).

11 Viswanathan, S. R. *et al.* Lin28 promotes transformation and is associated with advanced human malignancies. *Nat Genet* **41**, 843-848, doi:ng.392 [pii]

10.1038/ng.392 (2009).

1. <http://www.aocstudy.org/> [↑](#footnote-ref-1)
2. <http://www.ncbi.nlm.nih.gov/geo/query/acc.cgi?acc=GSE9891> [↑](#footnote-ref-2)
3. <http://cancergenome.nih.gov/> [↑](#footnote-ref-3)
