# Supplementary material for: Deregulation of MYCN, LIN28B and LET7 in a Molecular Subtype of Aggressive High-Grade Serous Ovarian Cancers
Source: PLoS One. 2011 Apr 13;6(4):e18064. doi: 10.1371/journal.pone.0018064 (PMC3076323; doi:10.1371/journal.pone.0018064)
Supplement: Table S6 — Summary of FISH on TMA for AOCS samples of known molecular subtype. (PDF) [file pone.0018064.s012.pdf]

Supplementary Table 6: Summary of FISH on TMA for AOCS samples of known molecular subtype

	C5	Non-C5
Samples (n)	16	57
Normal Copy number at *LIN28B* / *HACE1* locus	12	39
Gain of *LIN28B* probe	1[*]	1[§]
Gain of *HACE1* probe	0	0
Gain of loci (both probes)	3	9
Loss of *LIN28B* probe	0	0
Loss of *HACE1* probe	0	0
Loss of Loci (both probes)	1[*]	9[§]
Re-arrangements (break apart of *HACE1* and *LIN28B* probes)	0	0

[*]Inconsistency between replicate cores for 1 C5 sample, one core showing gain of LIN28B probe and an independent core showing only a single copy of the LIN28B and HACE1 locus

[§] Inconsistency between replicate cores for 1 Non-C5 sample, one core showing gain of LIN28B probe and an independent core showing only a single copy of the LIN28B and HACE1 locus

	C5	Non-C5
Neutral Copy Number	12	39
Any Gain of Loci	4	10
Any Loss of Loci	1	9

Chi-square	
Chi-square, df	1.215, 2
P value	0.5446
